# Avatar therapy for persecutory auditory hallucinations: What is it and how does it work?

**DOI:** 10.1080/17522439.2013.773457

**Published:** 2013-03-04

**Authors:** Julian Leff, Geoffrey Williams, Mark Huckvale, Maurice Arbuthnot, Alex P. Leff

**Affiliations:** a University College London, Mental Health Sciences, 1 South Hill Park Gardens, London, NW3 2TD, United Kingdom; b University College London, Hearing, Speech and Phonetic Sciences, Chandler House, 2 Wakefield Street, London, WC1N 1PF, United Kingdom; c University College London, Speech, Hearing and Phonetic Sciences, Chandler House, 2 Wakefield Street, London, WC1N 1PF, United Kingdom; d University College London, Mental Health Sciences, Flat D, Bentley House, Kings Scholar Passage, London, SW1P 1NN, United Kingdom; e Institute of Cognitive Neuroscience, Brain Repair and Rehabilitation, Institute of Neurology, Queen Square, London, WC1N 3BG, United Kingdom

**Keywords:** hearing voices, treatment outcome research, child abuse, client-centred therapy, defence mechanisms, avatar therapy

## Abstract

We have developed a novel therapy based on a computer program, which enables the patient to create an avatar of the entity, human or non-human, which they believe is persecuting them. The therapist encourages the patient to enter into a dialogue with their avatar, and is able to use the program to change the avatar so that it comes under the patient's control over the course of six 30-min sessions and alters from being abusive to becoming friendly and supportive. The therapy was evaluated in a randomised controlled trial with a partial crossover design. One group went straight into the therapy arm: “immediate therapy”. The other continued with standard clinical care for 7 weeks then crossed over into Avatar therapy: “delayed therapy”. There was a significant reduction in the frequency and intensity of the voices and in their omnipotence and malevolence. Several individuals had a dramatic response, their voices ceasing completely after a few sessions of the therapy. The average effect size of the therapy was 0.8. We discuss the possible psychological mechanisms for the success of Avatar therapy and the implications for the origins of persecutory voices.

## The problem to be tackled

One in four people who hear persecutory auditory hallucinations fails to respond to antipsychotic medication, with a severe impairment to their quality of life.

Two observations engendered the concept of avatar therapy. When people are asked about the worst aspect of hearing voices, their invariable response is helplessness. However, people who can establish a dialogue with their voices feel much more in control (Nayani & David, 1996). It was our intention to devise a method of facilitating a dialogue between the voice hearer and the entity they believe to be speaking to them. Attempting to maintain a dialogue with an invisible entity is difficult for several reasons. Hearing a disembodied voice abusing you in stereotyped phrases taxes your resources as a social human being. Because the entity is invisible there are none of the usual cues of facial expression and non-verbal communication by which we signal agreement with, attention to, and turn-taking with the speaker. The method we chose to initiate a genuine conversational interchange was to enable the patient to create an avatar of the entity, human or non-human, and to encourage them to engage in a dialogue with the avatar. The primary aim of the therapy was to facilitate the dialogue so that with the therapist's encouragement the patient would learn to stand up to their avatar and eventually control it. It was hoped that this experience would generalise to the persecutory voice.

### The nature of the computerised system

Two commercial programs were included in a package in conjunction with a unique voice-morphing program developed by one of us (MH). The patient constructs the avatar, using the software to choose a face and a voice that approximates to the entity they hear. The range of voices from which the patient chooses is produced by morphing the therapist's voice into a variety of forms. This enables the therapist to speak to the patient through the avatar in real time using the selected voice.

### The physical set-up of the system

The patient sits in a room and faces a monitor on which their avatar is shown. The avatar's lip movements are synchronised with its speech by the software. The therapist sits in an adjacent room and views a screen. Clicking on the right side of the screen allows the therapist to speak to the patient through the avatar using the morphed voice. Clicking on the left side of the screen enables the therapist to speak to the patient in their normal voice.

### The interaction between the patient and their avatar

The patient is prompted to enter into a dialogue with their avatar and encouraged to oppose it. The therapist controls the avatar so that it gradually comes under the patient's control over 6 weekly sessions of 30 min duration. Over the course of the therapy the avatar progressively changes from being persecutory to becoming appreciative and supportive. Each session is digitally recorded and the audio file transferred to a personal media player which is given to the patients to use at any time to reinforce their control over the persecutory “voice”.

## Efficacy of the therapy

Avatar therapy was compared with treatment as usual in a randomised controlled trial with a crossover of the control group from treatment as usual into Avatar therapy after an initial 7-week block. Treatment as usual comprised antipsychotic medication, which was taken regularly by all but two of the participants, and regular appointments with the clinician responsible for their care. Informed consent was obtained from all participants. The duration of hearing voices ranged from 4 to more than 30 years, the median being more than 10 years. Patients were assessed on three occasions: at baseline, 1 week after the therapy block, and 3 months later, the latter two assessments being conducted by an independent blind assessor. The assessment instruments were the Psychotic Symptom Rating Scale (PSYRATS) hallucinations score (Haddock, McCarron, Tarrier, & Farragher, 1999), the BAVQ-R scores on omnipotence and malevolence (Chadwick, Lees, & Birchwood, 2000), and the Calgary Depression Scale (Addington, Addington, & Mticka-Tyndale, 1993).

Of the 26 patients who entered the trial, 16 received the therapy, and benefitted from significant reductions in the frequency and intensity of the voices and in the disturbance to their life. There was also a significant amelioration in the perceived malevolence and omnipotence of the voices. At the 3-month follow-up there were further reductions in the frequency and intensity of the voices. Additionally, a significant reduction in depressive symptoms was detected when scores at the end of therapy were compared with the 3-month follow-up assessment. The average effect size of the therapy was 0.8. A full account of this pilot study will appear in the *British Journal of Psychiatry* (Leff et al., in press).

The most dramatic effects were experienced by three patients who had been hearing voices incessantly for 16 (A), 13 (B), and 3.5 years (C). A had stopped taking medication 3 years previously. B and C took adequate antipsychotic medication regularly. Patients A and C ceased to hear their “voice” after the second session, each receiving a total of 1 hour of therapy, while Patient B's “voice” ceased after the fifth session. The voices of all three patients were still absent at the 3-month follow-up. The brevity of Avatar therapy and its success in decreasing the frequency of the voices, their volume, and their impact on patients' lives requires an exploration of the possible mechanisms for these dramatic effects on experiences which have failed to respond adequately to antipsychotic medication.

## Possible mechanisms of action: two strategies

JL, the sole therapist in this trial, had a clear idea of what he was aiming for, and as he gained experience of what worked and what did not, he jettisoned some strategies borrowed from Cognitive Behaviour Therapy, and developed some new ones, particularly to help people with very low self-esteem, which emerged as a common factor among the voice hearers. Strategies which proved ineffective were: encouraging people to set a particular time of day or night when they would listen to their voices, and telling the avatar that they would only listen when (s)he said pleasant things. JL employed two quite different strategies according to the degree of insight possessed by the person. If they lacked insight completely, he accepted the reality of the avatar for the person and dealt with it on that basis. An example is the elderly man (C) who had been a senior executive in a large company. Several years before he was inducted into the trial, he began to be woken every morning at 5 a.m. by the voice of a woman, also a senior company executive. She held business meetings from that early hour until nightfall, so that he heard her discussing business matters with her subordinates throughout the entire day, although she never addressed him directly. He was completely convinced of the reality of this woman, but had not developed an explanation for her disembodied voice. JL accepted the patient's experience as real and advised him that the woman was behaving unprofessionally, and that he should tell the avatar to confine her meetings to business hours. Furthermore, she was betraying her organisation by letting him hear her discussions. In the first session with the avatar C was polite, spoke in a soft voice and remained calm and quiet throughout the session, which was ended after 15 min. One week later he arrived for the second session and reported that her voice was quieter, as were the voices of her subordinates. Also they started at 8 a.m. instead of 5 a.m. In this session C was more forceful, and told the avatar that he did not want to hear her plans, saying, “It's treason. Keep it to yourself. He told her to confine her meetings to the afternoon, after 2 p.m. He said, “I don't want to hear you at 8 a.m. I have a lot to do in the mornings and you disturb me.” In general he was much more assertive than in the first session. When C arrived for the next session he reported that he was sleeping until 7 a.m. and that the woman's voice had gone entirely, “as though she left the room”. At a follow-up 1 week later the woman's voice was still absent, and had not resumed at a 3-month follow-up.

C was the fourth patient to receive the therapy, and we were taken by surprise at the cessation of his “voice” after only two sessions. We were cautious, thinking this might be a one-off, but later on other patients ceased to hear their persecutors.

**Figure 1.**
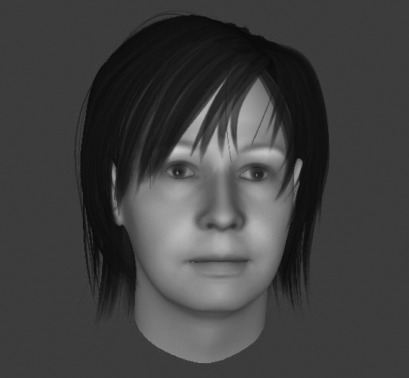
Avatar of patient D.

An alternative strategy was employed by JL with patients who had some degree of insight into the origin of the voice as being in their own mind. An example of this is D, the patient with the longest duration of hearing voices: over 30 years. As a child he lived with his mother and two older brothers who bullied him. Their mother went to the pub every evening leaving her sons in the care of an alcoholic man. D heard several different voices, but as we could only work with one voice as an avatar, we asked patients which voice was dominant, or if none was, which voice they would rather be without. D chose the voice of a woman who made sarcastic, unhelpful comments, such as “Playing you and you've been really ill. Insane at least because you're totally out of your skull as well as out of your face”. (Figure 1) D said that he believed that the sentences came from thoughts in his head, indicating a considerable degree of insight. JL focused on this during the sessions.

Physical abuse, sexual abuse and emotional neglect have been linked by several researchers with the later development of schizophrenia (Bebbington et al., 2011; Hussey Chang, & Kotch, 2006; Read, Agar, Argyle, & Aderhold, 2003; Schenkel, Spaulding, DiLillo, & Silverstein, 2005). D had made the link between his childhood trauma and the persecutory voices. JL explained that he could not put his feelings into words as a young child. Now he can begin to understand why he is denigrating himself through the voices. D worked as a volunteer in a charity shop and valued his position. He was listening regularly to the recorded sessions on the personal media player and said, “You've given me tools to get rid of the voices, but I don't understand much of it”. The avatar agreed to say only pleasant things to him and JL asked him what the good things about him were. He replied that he does his job, he is punctual, and has friends. He was prompted to tell the avatar that he will only listen to nice commands, not to bad ones. The avatar promised to say nice things to D. At this stage in the development of the therapy, JL had not worked out a more effective way of increasing patients' self-esteem, which for many of them was pathologically low, being reflected in the content of the persecutory voices. Later, JL introduced another strategy. He asked the patient who was closest to him or who knew him best. Once this person was identified, JL asked the patient to request that person to make out a list of the patient's good qualities. When the list had been compiled, the patient was instructed to bring it to the next session. The avatar then went through the list with the patient asking him to confirm each good quality and congratulating him on it. This dialogue was as usual recorded on the personal media player and the patient was encouraged to listen to this recording whenever he felt low in mood.

D showed considerable improvement at the follow-up 1 week after the end of six sessions of therapy. The voices reduced from being continuous to occurring once a week for several minutes. Instead of being mostly unpleasant, they were only occasionally so. From believing more than 50% that the voices came from an external source, D changed to considering them entirely self-generated. His self-depreciation reduced from severe to mild, and suicidal thoughts from mild to absent. At his 3-month follow-up, self-depreciation was absent. This can be attributed to the planned change in the avatar's relationship to the patient, altering from continual denigration to a pleasant supportive role. It was evident that the patient's avatar was a composite of his bullying older brothers and his neglectful, uncaring mother. As the avatar ceased her punitive verbal attacks and expressed admiration of his good qualities, which he had never before experienced, the persecutory voices became much less frequent and rarely critical. His image of himself improved in parallel with these changes in his experience with the result that over time he became able to appraise himself as worthwhile and lost any thoughts of suicide.

## Nature of the dialogue

It is in fact a trilogue because the therapist plays two roles, the persecutory avatar and the supportive therapist. As the sessions proceed the two roles merge and the avatar progressively agrees to stop abusing the patient and begins to make helpful suggestions and to boost the patient's self-esteem. In accord with this, the avatar's expression is changed from menacing or neutral to smiling. Although the patients interact with the avatar as though it were a real person, because they created it they know that it cannot harm them, as opposed to the voices, which often threaten to kill or harm them and their family. They can take risks with the avatar, standing up to it and telling it forcefully to leave them alone, behaviour they would not dare to attempt with the persecutor for fear of reprisals. Once they gain the courage to oppose the avatar, they learn to do the same with their persecutor (see patient E below). Some patients, particularly young women who have been sexually abused, are very timid when they are faced with their avatar for the first time and speak very softly, requiring considerable encouragement from the therapist to speak louder and defend themselves vigorously. A minority of patients have found it impossible to proceed with the therapy. Two women who heard multiple voices speaking very loudly could not concentrate on what the avatar was saying and abandoned the therapy after the second session. A man who believed he was being persecuted by the devil complained that whenever he tried to create the avatar, the devil gave him severe pains in his groin, and he dropped out of therapy.

Patient A, referred to above, was a financier who, 16 years previously, began to hear the devil giving him advice on investments. He created the avatar as a red-faced devil and was fully convinced of the reality of this being. He took the devil's advice, lost all his money, and was heavily in debt. JL treated the devil avatar as a real entity, and encouraged the patient to tell the devil to leave him alone and go back to Hell where he belonged. A was very vigorous in attacking the avatar both in the first and second sessions. When he arrived for the third session he reported that after the second session, as he was walking away from the hospital, the voice began to speak to him. He said firmly to the voice, “You are not coming back”, and from that moment on the voice ceased. He felt his depression lifting and said his life had changed dramatically. He thanked us for giving him his life back. At the 1-week follow-up the voice was still absent, but at the 3-month follow-up he said he had suffered a slight relapse. The voice was absent during the day, but had come back at night. JL asked him how he spent his evenings and he replied that he worked on his computer until 1 or 2 a.m. Also he had not been using his personal media player. JL told him that he was still vulnerable and should not overstrain himself. He advised A to go to bed by 12 p.m. and to listen to his personal media player before falling asleep. At a follow-up 2 weeks later, A said that the voice had ceased and that his head was completely clear, which he attributed to the therapy.

## Possible explanations for the effectiveness of the therapy

### Face validity of the patient's experience

In the case of patients A and C, JL and GW accepted the patients' experience of the person or entity persecuting them. They assisted the patients to create their avatar, asking throughout the procedure how close a match there was between the image on the monitor or the voice they had selected and what they believed their persecutor looked and sounded like. This confers face validity on the patient's experience. Many clinicians view this approach as collusion with the patient's pathology and likely to perpetuate the symptoms. Some therapists encourage patients to speak to an empty chair as though they were addressing their persecutor (e.g. Corstens et al.) Avatar therapy goes a step further in that the therapist assists the patient to create a speaking image of their tormentor which the therapist can also see and hear. Because the externalised voice is part of the patient's inner world, discounting it or refusing to acknowledge the patient's experience of this split-off part as real negates the possibility of the patient reintegrating it into their psychic structure.

### The effect of establishing a dialogue with the avatar

Because the avatar's speech is emitted by a human being (the therapist) in real time, it is convincingly responsive to what the patient has just said. Thus a realistic dialogue is engendered. (An audio clip of a session with a patient can be accessed at http://www.phon.ucl.ac.uk/home/geoff/avatar/demos/010-S1-clip.mp3.)

This is not necessarily the case in the first session when the avatar utters the brief phrases that the patient hears from their persecutor. By the second session, having established its identity, the avatar expands its responses to facilitate a normal conversational interchange, giving the patient hope that they can negotiate with the avatar. Almost all the 16 patients who received the therapy behaved toward the avatar as though it was a real person. E, a woman of 41, was convinced that the voices tormenting her had an external reality. She was encouraged to oppose the voices and when she came for her third session she said, “The voices are milder 'cos they know that if they talk a lot, I'll say something”.

### Patients' relationship with the avatar

Two of the 16 did not accept the avatar as a representation of their hallucination. One, a male student, addressed his remarks to JL by name instead of to the avatar. The other was an adolescent girl who heard multiple voices and saw images of people from whom she believed the voices emanated. These experiences had led to a diagnosis of borderline personality disorder by her psychiatrist. However, JL was convinced that this condition coexisted with schizophrenia, a recognized comorbidity (Thérien, Tranulis, Lecomte, & Bérubé, 2012). After the first session, when JL entered the room she was in to ask her for feedback, she blurted out angrily, “It's a fake!” JL responded that there was no attempt to pretend that the avatar was really the person whose voice she heard. The avatar was her creation, and therefore it was safe for her to try out different strategies to counter its abuse, something she did not dare to do with the voices she heard. The male student benefitted very little from the therapy, and the adolescent girl not at all.

### The avatar modifies its character over time

An important component of the therapy is that after the first two or three sessions the avatar is made to cease being abusive and controlling, and becomes increasingly supportive of the patient, complimenting them on their achievements, suggesting ways in which they could improve their life, and praising their good qualities. In accord with this change in character, the avatar's expression is altered to appear friendly and smiling. This may be appraised subconsciously by the patient as a substitution of a loving parent for a punitive, denigratory, or neglectful one, enabling them to reintegrate the projected unacceptable part of their internal world into their psychic structure. In a similar vein, Garrett and Turkington (2011) argue that CBT provides a technique to bring “thing presentations” (thoughts or feelings experienced as an external perception) back within the boundary of the self.

### Helping patients to overcome fear of their persecutors

Many patients fear to oppose their persecutors, who threaten to harm them or their family if they disobey. One patient in our trial who heard his dead grandmother's voice abusing him declined to accept the therapy because his grandmother forbade him to take part. The therapy provides patients who are determined enough to proceed with it the opportunity to try out various strategies of opposing the avatar with no fear of reprisals against them or their relatives. Patients, mostly young women, who are very timid in their first encounter with their avatar and speak in a barely audible voice, are reminded that the avatar is only a representation of their persecutor that they themselves have created and is therefore incapable of harming them. The message is reinforced that what proves effective with the avatar can then be attempted with their persecutory voices.

### The experience of gaining control over the avatar

As the avatar becomes less dominating and the therapist encourages the patient to be increasingly forceful in opposing it, the patient gains confidence in their power. Hayward and colleagues (2011) have reviewed the evidence that the degree of perceived dominance of the voices directly reflects the patient's subordinate position in relation to their social contacts. Being low down in the social pecking order may derive from the patient's treatment in their family of origin, as with patient D. Avatar therapy, by enabling the patient to achieve control over their avatar, leads to a reduction in their feeling of helplessness and enables them to face their actual persecutor with increased courage and boldness. This is exemplified by patient A, who responded to the voice of the devil by saying. “You are not coming back!”

### Making the patient aware of the link between their low self-esteem and the critical statements of the voices

Both the therapist and the avatar, in its benevolent persona, link the patient's low self-esteem with the abuse from the voices. The avatar comments that “the voices say what you think about yourself.” This helps the patient to recognise that the voices originate from within his/her own mind. Garrett and Turkington (2011) employ a similar approach formulated as: “We have discovered through our investigations that the critical voices who say you are worthless are not all-powerful authorities as you once believed, but a way you experience your own self-critical thoughts.” The Avatar therapist tells the patient that if he begins to acknowledge his own good qualities, this will ameliorate the abuse. Our strategy of requiring the patient to ask a close friend or relative to write down a list of his or her good qualities constitutes the first step in attempting to raise the patient's self-esteem. This is taken to the next level when the avatar goes through the list item by item, asking the patient to confirm what the informant has written down, and congratulating her or him on each quality. The whole sequence in which the patient is faced with these valued aspects of the self challenges their negative view of their nature, and can be accessed repeatedly by using the personal media player. We believe this partially explains the reduction in depression which occurred after the end of therapy.

## Sexual abuse and psychotic experiences

A substantial body of research has accumulated linking sexual abuse in childhood or adolescence with the later development of psychosis (e.g. Bebbington et al., 2011; Read, Fink, Rudegaier, Felitti, & Whitfield, 2008). Four of the 26 patients in the trial had been sexually abused in childhood or adolescence. Irons, Gilbert, Baldwin, Baccus, and Palmer (2006) found that recall of parents as being rejecting and overprotective was significantly related to both inadequacy and self-hating criticism. This may explain why three of our four abused patients heard voices blaming them for the rape. For two of these patients, JL used the avatar to help reduce their sense of responsibility for the abuse. This was successful for one of them who listened to his recorded voice on the player, saying, “It wasn't my fault.”

## Advantages of the personal media player

The personal media player which is given to each patient to keep contains all their recorded sessions. Both the therapist and the avatar encourage the patients to listen to their personal media player when harassed by the voices. We describe it to them as “a therapist in their pocket”. Being portable, the personal media player is available to the patients night and day. Patients can record their own choice of music on the personal media player and can use that or the recorded dialogues with their avatar to override the voices. The recorded sessions also remind them of their success in standing up to the avatar, and bolster their courage in opposing the voices. The particular sessions in which the avatar reviews the list of the patient's good qualities act as useful reminders to the patient that someone they trust appreciates them, and probably help to elevate the patient's self-esteem. The continued diminution of auditory hallucinations after therapy ended, and the significant reduction in depressive symptoms over the whole period of the follow-up, can probably be ascribed to the availability and regular use of the personal media player, although we did not collect accurate data on this. This hypothesis will be tested in the large-scale trial to follow.

## Discussion

Avatar therapy has proved to be an efficacious treatment for most patients with persecutory auditory hallucinations that have not responded to antipsychotic medication, who are willing to participate in the therapy. For a small number of patients it has the dramatic effect of abolishing their “voices” altogether, even after many years of being dominated by them. It is brief, no more than 7 sessions of up to 30 min, and therefore economical of therapists' time. It is not acceptable to every potentially suitable patient: out of 26 patients who entered the trial, 4 refused the therapy when it was offered, and 5 dropped out before receiving three sessions, the minimum set as an adequate therapeutic input. It is clearly essential to determine whether an independent team of researchers and therapists can replicate these promising findings on a much larger sample, and a grant has recently been awarded for such a study, with a proposed sample size of 142.

### Clinical implications

In addition to its therapeutic potential, Avatar therapy offers us the experience of directly observing patients interacting with a representation of their “voices”. This illuminated many aspects of the patients' relationship with their “voices” which are discussed above, including the important psychodynamic formulation of the externalisation of unacceptable thoughts, and the possibility of reversing this process. As it evolved, Avatar therapy incorporated a variety of different strategies, both practical and theoretical. In order for others to master this therapy, it is necessary to construct a treatment manual and this has now been completed, in preparation for the replication study. One of its main aims is to determine whether clinicians working in a standard setting can be trained to achieve results comparable to those that emerged from the pilot study.
